# Association of initial prednisolone dose with remission, relapse, and infectious complications in adult-onset minimal change disease

**DOI:** 10.1007/s10157-021-02119-3

**Published:** 2021-08-07

**Authors:** Kaori Tanabe, Ken-ichi Samejima, Fumihiro Fukata, Takaaki Kosugi, Hideo Tsushima, Katsuhiko Morimoto, Keisuke Okamoto, Masaru Matsui, Masahiro Eriguchi, Naoki Maruyama, Yasuhiro Akai, Kazuhiko Tsuruya

**Affiliations:** 1 Department of Nephrology, Nara Medical University, 840 Shijo-cho, Kashihara, Nara 634-8521 Japan; 2 Department of Community-Based Medicine, Nara Medical University, Nara, Japan; 3 Department of Nephrology, Nara Prefecture General Medical Center, Nara, Japan; 4 Department of Nephrology, Nara Prefecture Seiwa Medical Center, Nara, Japan; 5 Department of Nephrology, Saiseikai Suita Hospital, Suita, Japan

**Keywords:** Minimal change disease, Prednisolone, Remission induction, Recurrence, Cumulative steroid doses

## Abstract

**Background:**

A dose of 0.5–1 mg/kg/day of prednisolone (PSL) is administered for the initial treatment of minimal change disease (MCD). However, little is known about the optimal PSL dose for the initial treatment of MCD.

**Methods:**

We conducted a retrospective multicenter cohort study of treatment-naive adult patients with MCD diagnosed by renal biopsy from 1981 to 2015 in whom PSL monotherapy was performed as the initial treatment. The exposure of interest was an initial median PSL dose of < 0.63 mg/kg/day (Group L) compared to ≥ 0.63 mg/kg/day (Group H). Cumulative remission and relapse after remission were compared between these groups using Cox regression adjusted for baseline characteristics.

**Results:**

Ninety-one patients met the inclusion criteria. During a median follow-up of 2.98 years, 87 (95.6%) patients achieved complete remission, and 47.1% relapsed after remission. There was no significant difference in the remission rate between the groups at 4 weeks of follow-up (66.7 vs. 82.6%). The median time to remission in Group L was comparable to that in Group H (17.0 vs. 14.0 days). A multivariable Cox hazard model revealed that the initial PSL dose was not a significant predictor of remission. The cumulative steroid doses at 6 months, 1 year, and 2 years after treatment initiation were significantly lower in Group L than in Group H.

**Conclusion:**

The initial PSL dose was not associated with time to remission, remission rate, time to relapse, or relapse rate. Therefore, a low initial steroid dose may be sufficient to achieve remission.

**Supplementary Information:**

The online version contains supplementary material available at 10.1007/s10157-021-02119-3.

## Introduction

In most cases, minimal change disease (MCD) responds to steroid therapy, and more than 90% of cases achieve a complete remission with steroid therapy [1, 2]. However, as a treatment, it is problematic as 30–70% of cases relapse [1, 3–5]. The Kidney Disease Improving Global Outcomes (KDIGO) guidelines recommend prednisolone (PSL) at an initial daily single dose of 1 mg/kg (maximum 80 mg) or alternate-day single doses of 2 mg/kg (maximum 120 mg) for the treatment of MCD [6]. These are supplemented by the 2014 Guidelines for Nephrotic Syndrome in Japan, which recommend an initial dose of PSL ranging from 0.8 to 1 mg/kg (maximum 60 mg) [7]. However, little is known about the optimal PSL dose for the initial treatment of MCD, with initial doses of PSL ranging from 0.5 to 1 mg/kg/day [1, 3–5].

In patients who relapse in our institution, we often prescribe lower steroid doses than the dose at first onset and have observed that most patients with relapse can achieve remission quickly. Considering the objective of preventing infections associated with steroid treatment, lower doses of steroids are desirable. Therefore, it was hypothesized that low initial doses of steroids could be as effective as high initial doses for inducing remission but would be associated with fewer infections. To the best of our knowledge, in adult-onset MCD, there are no reports of an association between the initial dose of oral PSL and prognosis. Here, we retrospectively examined patients with MCD treated initially with steroids at our department and at four related institutions.

## Materials and methods

We conducted a retrospective, multicenter, cohort study of treatment-naive adult patients with MCD diagnosed by renal biopsy from 1981 to 2015 in Nara Medical University Hospital, Nara Prefecture General Medical Center, Nara Prefecture Seiwa Medical Center, Saiseikai Suita Hospital, and Municipal Oyodo Hospital. Written informed consent was not required because of the retrospective observational study design. Instead, we offered the patients the right to opt out. This study was approved by the Nara Medical University Ethics Committee (No. 1054).

### Inclusion/exclusion criteria

Inclusion criteria were as follows: (1) diagnosis of MCD; (2) > 15 years of age; and (3) initial treatment with steroids alone. Exclusion criteria were as follows: (1) missing clinical records; (2) fewer than 10 glomeruli seen on renal biopsy; (3) adult-onset MCD but not first-onset MCD; (4) remission before steroid treatment; (5) other diseases treated with PSL at the time or previously; (6) other comorbidities (e.g., active cancer, Kimura’s disease, hepatic insufficiency); or (7) poor drug compliance that we could confirm from medical records with certainty.

### Renal biopsy

Renal biopsy specimens were collected at the Nara Medical University Hospital. MCD was defined as nephrotic syndrome at the time of consultation, with glomeruli showing almost normal changes on light microscopy and the absence of staining on immunofluorescence. Histologic examinations were performed independently by at least two renal nephrologists.

### Baseline characteristics

Baseline characteristics were recorded from medical records retrospectively, including data recorded before renal biopsy and before starting steroid treatment. Data included sex, age, body weight (BW), body mass index (BMI), blood pressure, serum creatinine, eGFR (mg/day/1.73 m^2^), serum albumin, proteinuria, total cholesterol, and initial PSL dose (mg/day and mg/kg/day). The eGFR was calculated for adults aged 18 years or older using a formula modified for Japanese adults [8], and for children aged less than 18 years using a formula modified for Japanese children [9]. Based on previous reports [10], we calculated that 1 mg of methylprednisolone (mPSL) was equivalent to 1.25 mg of PSL.

### Outcome

The exposure of interest was a median initial dose of PSL < 0.63 mg/kg/day (Group L) compared to ≥ 0.63 mg/kg/day (Group H). We retrospectively analyzed the complete remission rate, the time to first remission from treatment initiation with PSL, relapse rate, the time to first relapse from first complete remission, complications (i.e., acute kidney injury [AKI] and incidence of infection requiring hospitalization), death, and cumulative PSL dose in Group H and Group L.

We defined complete remission as a daily urinary protein level of less than 0.3 g/day or less than 0.3 g/g Cr in accordance with the 2014 Guidelines for Nephrotic Syndrome in Japan [7]. Relapse was defined as two or more occasions of elevated urinary protein or when the physician in charge determined that the disease had relapsed and the PSL dose was increased. AKI was defined as increased serum creatinine more than 1.5 times the baseline serum creatinine value (minimum serum creatinine during the treatment course). The total observation period was from the start of steroid treatment until the final observation day.

### Statistical analysis

Statistical calculations were completed using JMP 14.2.0 (SAS Institute Inc., Cary, NC, USA). Data are expressed as medians (25th and 75th percentiles). Cumulative remission and relapse after remission were compared using Cox regression adjusted for sex, age (per 1 year), estimated glomerular filtration rate (per 1 mL/min/1.73 m^2^), and proteinuria (per 1.0 g/day), and using Kaplan–Meier curves. Median time to remission, remission rate, median time to relapse, relapse rate, incidence of adverse events during a set time period following steroid therapy, and cumulative dose of PSL were also compared using the Mann–Whitney *U* test and the chi-square test. Values of two-tailed *p* < 0.05 were considered significant.

## Results

**Fig. 1:**
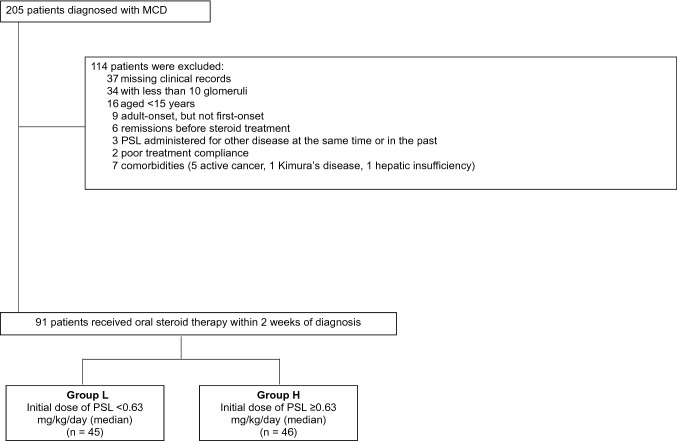
Patient inclusion. MCD was diagnosed in 205 patients between 1981 and 2015. Of these, 91 patients met the inclusion criteria. MCD, minimal change disease; PSL, prednisolone

MCD was diagnosed in 205 patients between 1981 and 2015. Of these, 91 patients met the inclusion criteria (Fig. 1). Baseline characteristics are summarized in Table 1. Forty-eight patients (52.7%) were male, and the total median age was 46 years. The median BW was 62.3 kg, and the median initial PSL dose was 0.63 mg/kg/day. The median initial PSL doses were 0.52 and 0.76 mg/kg/day in Groups L and H, respectively. Across all participants, the median serum creatinine was 0.86 mg/dL, and the median proteinuria was 7.82 g/day. There were significant differences (*p* < 0.05) between Groups L and H with regard to sex, BW, BMI, serum creatinine, systolic blood pressure, serum albumin, and initial PSL dose (Table 1).

**Table 1:** Baseline characteristics at the initiation of corticosteroid treatment

	Total	Group L	Group H	*p* value
	(*n* = 91)	(*n* = 45)	(*n* = 46)	
Male, *n* (%)	48 (52.7)	31 (68.9)	17(37.0)	0.002
Age, years	46 [29–65]	48[29.5–68]	43 [26.8–63.3]	0.46
Entire observation period, years	2.98 [1.71–5.10]	3.36 [1.84–5.02]	2.61 [1.57–6.68]	0.86
Body weight, kg	62.3 [53.0–74.5]	73.0 [65.0–79.5]	54.6 [49.9–59.1]	< 0.001
Body mass index, kg/m^2^	23.9 [21.0–26.9]	26.6 [24.0–28.0]	21.2 [20.0–23.8]	< 0.001
Serum creatinine, mg/dL	0.86 [0.71–1.10]	0.90 [0.80–1.14]	0.80 [0.60–1.10]	0.04
Systolic blood pressure, mmHg	120 [110–132]	128 [118–134]	119 [104–130]	0.02
Diastolic blood pressure, mmHg	74 [70–80]	75 [69–80]	74 [69–80]	0.69
eGFR, mL/min/1.73 m^2^	68.7 [56.0–88.7]	65.1 [52.5–83.1]	74.0 [56.3–89.8]	0.29
Serum albumin, g/dL	1.9 [1.6–2.3]	1.8 [1.6–2.2]	2.0 [1.8–2.4]	0.04
Proteinuria, g/day	7.8 [5.7–12.0]	8.4 [6.0–12.3]	7.1 [5.0–12.1]	0.20
Total cholesterol, mg/dL	433 [328–519]	449 [374–561]	415 [317–492]	0.07
Initial PSL dose^a^, mg/kg/day	0.63 [0.52–0.76]	0.52 [0.47–0.60]	0.76 [0.70–0.84]	< 0.001
PSL 20/30/40/45/50/60/ mPSL 200/500/1000, mg/day, *n*	5/10/68/1/1/1 1/1/3	5/9/30/1/0/0 0/0/0	0/1/38/0/1/1 1/1/3	0.009

Values are given as *n* (%) or median [interquartile range], as appropriate

eGFR, estimated glomerular filtration rate; PSL, prednisolone; mPSL, methylprednisolone

^a^Methylprednisolone 1 mg was considered equivalent to prednisone 1.25 mg

### Complete remission

**Fig. 2:**
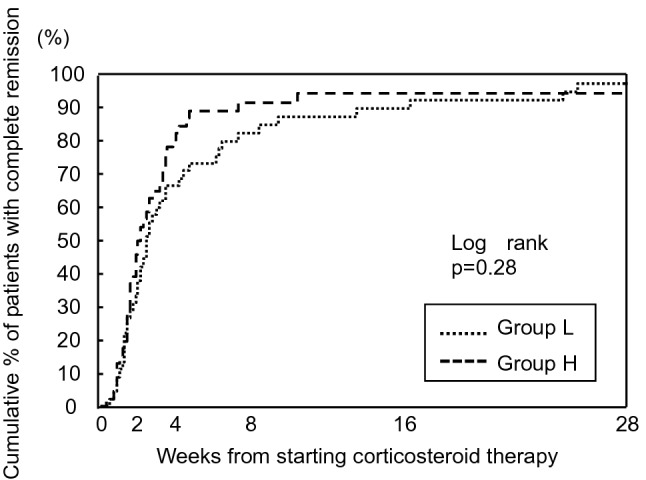
Cumulative percentage of patients with complete remission according to the initial steroid dose. There was no difference in time to remission between Groups L and H according to the results of Kaplan–Meier analysis

During a median follow-up of 2.98 years, 87 (95.6%) patients achieved complete remission. The median time to remission was 15 days. We examined the therapeutic effect of the initial steroid dose. There were no significant differences in the remission rates between Groups L and H at 4 weeks (66.7% vs. 82.6%), 8 weeks (82.2% vs. 91.3%), or 16 weeks (88.9% vs. 93.4%). Furthermore, the median time to remission in Group L was comparable to that in Group H (17 days vs. 14 days; Supplemental Table 1), and there was no significant difference in the time to remission according to the Kaplan–Meier analysis results (Fig. 2). Multivariable Cox hazard analysis revealed that the initial dose of PSL was not a significant predictor for remission [hazard ratio (HR) 0.83, 95% confidence interval (CI) 0.51–1.33; Table 2].

**Table 2:** Hazard ratios with 95% confidence intervals from multivariable regression analyses of the association of the initial dose of prednisolone (Group L versus Group H) with remission

Variables included in the regression models	HR (95% CI)	*p* value
*Crude*	0.80 (0.82–1.92)	0.30
*Model 1*		
Gender, age (per 1 year)	0.80 (0.51–1.28)	0.36
*Model 2*		
Model 1 + serum creatinine (mg/dL), proteinuria (per 1.0 g/day)	0.83 (0.51–1.33)	0.44
*Model 2’*		
Model 1+ eGFR (per 1 mL/min/1.73 m^2^), proteinuria (per 1.0 g/day)	0.82 (0.51–1.31)	0.40

HR, hazard ratio; CI, confidence interval

### Relapse

Of the 87 patients in complete remission, 41 (47.1%) relapsed. The median time to first relapse from first remission was 15.1 months. There was no significant difference in the relapse rate between Groups L and H (45.4% vs. 48.8%, *p* = 0.75). There was no significant difference in the time to first relapse from first remission between Groups L and H (16.5 months vs. 13.7; Supplemental Table 2). Similarly, no significant difference was observed on multivariable Cox hazard analysis (HR 0.87, 95% CI 0.44–1.75; Supplemental Table 3). There was no difference in the time to relapse from complete remission according to the Kaplan–Meier analysis results (Supplemental Fig. 1). There was no significant difference between the groups in terms of the dose of PSL at the time of relapse (Supplemental Table 2).

### Complications

Fifty-two (57.1%) patients experienced AKI, 10 (11.0%) patients developed infections that required hospitalization, and 6 (6.6%) patients died. Three of the six patients died within 6 months: one died of sepsis and one died of aspiration pneumonia in Group H, while one died of infectious pneumonia in Group L. Neither patient was in remission. The remaining three patients died more than 2 years later. The cause of death was acute myocardial infarction in one patient, and unknown in two patients who died suddenly. One patient, who had died suddenly of an unknown cause, was in his 30 s, but the rest were aged 70 years or older. There was no significant difference in the 6-month incidence of infectious complications requiring hospitalization between the groups (Group L: 7.3% vs. Group H: 4.6%; Supplemental Table 4).

Similarly, in the 80 patients who could be observed for over 1 year after treatment initiation, there were no differences in the 1-year incidence of infectious diseases requiring hospitalization (Group L: 7.5% vs. Group H: 5.0%) or the 1-year mortality (Group L: 2.5% vs. Group H: 5.0%). Similar results were obtained when 63 patients were observed for over 2 years (Supplemental Table 4).

### Cumulative PSL dose

We investigated the cumulative dose of PSL at 6 months, 1 year, and 2 years after treatment initiation and found that the cumulative PSL dose per kg body weight was significantly lower in Group L than in Group H at all time points (Table 3). Specifically, in the 82 patients who were followed for 6 months, the median cumulative PSL in Group L and Group H was 59.3 mg/kg and 86.5 mg/kg, respectively. In the 77 patients followed for 1 year, the median cumulative PSL dose in Group L and Group H was 85.6 mg/kg and 127.1 mg/kg, respectively. In the 60 patients followed for 2 years, the median PSL cumulative dose in Group L and Group H was 115.5 mg/kg and 149.5 mg/kg, respectively.

**Table 3:** Cumulative dose of PSL according to the initial dose

	Total	Group L	Group H	*p* value
	(*n* = 91)	(*n* = 45)	(*n* = 46)	
At 6 months	(*n* = 82)	(*n* = 40)	(*n* = 42)	
Cumulative dose of PSL (mg)	4713 [3933–5221]	4219 [3508–5159]	4876 [4169–5372]	0.06
Cumulative dose of PSL (mg/kg)	74.5 [58.3–89.3]	59.3 [51.1–72.2]	86.5 [74.6–103.3]	< 0.001
At 1 year	(*n* = 77)	(*n* = 39)	(*n* = 38)	
Cumulative dose of PSL (mg)	6755 [5675–8348]	6728 [4915–7860]	7148 [5881–9143]	0.14
Cumulative dose of PSL (mg/kg)	109.1 [83.1–139.8]	85.6 [76.1–116.8]	127.1 [104.8–171.9]	< 0.001
At 2 years	(*n* = 60)	(*n* = 33)	(*n* = 27)	
Cumulative dose of PSL (mg)	8862 [6992–11682]	8400 [6381–10619]	9150 [7208–15787]	0.24
Cumulative dose of PSL (mg/kg)	134.3 [86.2–171.5]	115.5[80.5–144.8]	149.5 [119.4–226.6]	0.005

PSL, prednisolone

Values are given as *n* (%) or median [interquartile range], as appropriate

## Discussion

In the present study, there were no differences in the remission rate or time to remission between MCD patients treated with PSL with a lower initial dose and a higher initial dose. The Cox proportional hazards model analysis of the relationship between initial PSL dose (per 0.1 mg/kg/day) and first remission showed no significant difference, with an HR of 1.00 (0.99–1.00) (data not shown). We also found that there were no significant differences between the PSL dosage groups with regard to the dose of PSL at the time of relapse from complete remission or with regard to the relapse rate.

Previous studies demonstrated that the relapse rate was 30–70% and the time to relapse was 5–26 months [3–5]. In the present study, the relapse rate was 47.1%, and the median time to relapse was 15.1 months (Supplemental Table 2), both comparable to the results of previous studies. The PSL dose per kg BW at the time of relapse did not differ between the groups (Supplemental Table 2), and the cumulative PSL dose per kg BW at 6 months, 1 year, and 2 years was significantly lower in Group L (Table 3). In other words, there was no significant difference in remission and relapse between the groups; however, the cumulative PSL volume was significantly lower in Group L than in Group H.

The relationship between the initial dose of steroids and MCD has been reported by Shinzawa et al. as the difference between mPSL and PSL [10]. Methylprednisolone and prednisolone use was significantly associated with early remission and lower incidence of relapse compared with oral prednisolone use alone. Moreover, the initial dose of prednisolone (per 0.1 mg/kg) was not associated with remission or relapse according to a multivariate analysis of Cox proportional hazards. In contrast, our study analyzed a larger number of patients who were initially treated with oral prednisolone and was able to demonstrate that a lower initial steroid dose was sufficient to achieve a therapeutic effect. Patients who are concerned about cosmetic problems such as moon face and acne or who have underlying diabetes or psychosis can be reassured that treatment with a low initial steroid dose can be effective. However, the small sample size, short observation period, and lower doses of steroids did not prove to be effective in reducing complications, and there were no cases of fractures.

Most patients with pediatric nephrotic syndrome are considered to have MCD, and the duration of the initial administration of steroids in children with nephrotic syndrome was studied and reported [11–13]. For idiopathic nephrotic syndrome in children, 2-month steroid treatment and 6-month steroid treatment with similar initial doses (60 mg/m^2^/day) were compared. There were no differences in the time from the initiation of PSL to relapse and in the relapse rate between the two groups [11]. Similar protocols have also been compared in adults where a 2-month short-term steroid regimen resulted in a higher relapse rate and shorter time to relapse than the conventional steroid regimen with a similar initial therapy dosage (0.8–1.0 mg/kg/day) [14]. In adults, short-term dosing is not as effective as it is in children with regard to the relapse rates, and it may be more effective to reduce the initial PSL dose, prolong treatment, and then taper the dosage down.

In this study, we found that the cumulative steroid dosage was significantly lower in Group L than in Group H at 6 months, 1 year, and 2 years after treatment initiation. Higher cumulative steroid doses and current steroid doses are associated with higher rates of infection [15–17]. While there was no significant difference in infections between Group L and Group H, fewer cumulative steroids may lead to fewer future infections. It has been reported that fractures occur in 30–50% of patients on long-term steroids [18, 19]; however, no fractures were found in this study despite the fact that it was conducted over a period of several years. Since repeated relapses of MCD result in a higher cumulative lifetime steroid dose, it is desirable to use as little steroids as possible to prevent fractures.

We found no differences between Group L and Group H with regard to remission, relapse, infectious complications, AKI, and death. Our results suggest that an initial lower dose of steroid therapy for the treatment of MCD is as safe and effective as a higher dose for inducing remission. Additionally, the time to remission was similar to that of other previous reports, despite the lower initial dose of corticosteroids compared with them [2, 20–22]. Furthermore, a lower steroid dose at relapse than that administered at the first onset led to remission in most cases. This may be due to relapses being detected early by regular follow-up examination, leading to earlier treatment. MCD has a rapid onset and is often discovered early, and even if it is the first onset, it is likely that treatment will start early.

We also analyzed the therapeutic effect of the initial steroid dose without weight correction. We performed multivariable regression analyses of remission and relapse in three groups of prednisolone-equivalent steroids (less than 40 mg, 40 mg to 45 mg, and 45 mg or more), but found no significant difference between the HRs (95% CIs) (Supplemental Tables 5, 7). Likewise, prednisolone doses (per 10 mg) were also analyzed, but no significant differences were found (Supplemental Tables 6, 8).

In most of the included patients, the initial prednisolone dose was 40 mg. Therefore, it may be concluded that 40 mg of prednisolone is sufficient to treat even large patients without weight correction.

This study had several limitations. First, this was a retrospective study with relatively small sample size. For this reason, it is difficult to identify the most suitable PSL dose for MCD. Second, this study excluded individuals who received immunosuppressive therapy at the start of treatment; therefore, there may have been a bias toward not including patients with severe MCD. Third, because MCD patients experience early remission, some patients do not go to the hospital after remission. Therefore, they may not have noticed if there had been a relapse. Fourth, this was an observational study, and lower steroid doses may have been given to high-risk individuals. For these reasons, it is necessary to perform randomized clinical trials to confirm the efficacy of initial PSL treatment at small doses. Nevertheless, Group L did not experience lower effects, more complications or deaths than Group H, suggesting that lower initial PSL was not a significant deterrent for MCD treatment. Despite these limitations, this study is considered important because of the following strengths: it is the first study to examine initial steroid doses for the treatment of MCD and it is one of the few studies that investigated cumulative PSL dosage and PSL doses at relapse.

## Conclusion

The initial dose of PSL in patients with MCD was not associated with time to remission, remission rate, time to relapse, or relapse rate. Based on these results, a low initial dose of steroid may be sufficient to achieve remission in MCD.

## Supplementary Information

Below is the link to the electronic supplementary material.

Supplementary file1 (DOCX 88 kb)
