# Supplementary material for: Association of initial prednisolone dose with remission, relapse, and infectious complications in adult-onset minimal change disease
Source: Clin Exp Nephrol. 2021 Aug 7;26(1):29–35. doi: 10.1007/s10157-021-02119-3 (PMC8738364; doi:10.1007/s10157-021-02119-3)
Supplement: Supplementary file 1 — Supplementary file1 (DOCX 88 kb) [file 10157_2021_2119_MOESM1_ESM.docx]

**Title**

Association of initial prednisolone dose with remission, relapse, and infectious complications in adult-onset minimal change disease

**Authors**

Kaori Tanabe, MD*^,1,2^, Ken-ichi Samejima, MD, PhD*^,1^, Fumihiro Fukata, MD^1,3^, Takaaki Kosugi, MD^1,4^, Hideo Tsushima, MD^1,5^, Katsuhiko Morimoto, MD, PhD^3,4^, Keisuke Okamoto, MD^1,5^, Masaru Matsui, MD, PhD^1,3^, Masahiro Eriguchi, MD, PhD^1^, Naoki Maruyama, MD^3^, Yasuhiro Akai, MD, PhD^1,2^, Kazuhiko Tsuruya, MD, PhD^1^

*The first two authors contributed equally to this work.

**Affiliations**

^1^Department of Nephrology, Nara Medical University, Nara, Japan

^2^Department of Community-Based Medicine, Nara Medical University, Nara, Japan

^3^Department of Nephrology, Nara Prefecture General Medical Center, Nara, Japan

^4^Department of Nephrology, Nara Prefecture Seiwa Medical Center, Nara, Japan

^5^Department of Nephrology, Saiseikai Suita Hospital, Suita, Japan

**Corresponding author**

Ken-ichi Samejima

Department of Nephrology, Nara Medical University, 840 Shijo-cho, Kashihara, Nara 634-8521, Japan

Tel.: +81-744-29-8865

Fax: +81-744-23-9913

E-mail: ksame@naramed-u.ac.jp

**Supplemental Table 1.** Remission rate and time to first remission according to initial PSL dose

|  | Total | Group L | Group H | p value |
| --- | --- | --- | --- | --- |
|  | (n = 91) | (n = 45) | (n = 46) |  |
| First remission within 4 weeks, n (%) | 68 (74.7) | 30 (66.7) | 38 (82.6) | 0.08 |
| First remission within 8 weeks, n (%) | 79 (86.8) | 37 (82.2) | 42 (91.3) | 0.20 |
| First remission within 16 weeks, n (%) | 83 (91.2) | 40 (88.9) | 43 (93.4) | 0.44 |
| First remission within the entire observation period, n (%) | 87 (95.6) | 44 (97.8) | 43 (93.5) | 0.32 |

|  | Total | Group L | Group H | p value |
| --- | --- | --- | --- | --- |
| Of the cases in remission | (n = 87) | (n = 44) | (n = 43) |  |
| Time to first remission, days | 15 [10–25] | 17 [10.0–40.5] | 14 [10–23] | 0.13 |

PSL, prednisolone. Values are given as n (%) or median [interquartile range], as appropriate.

**Supplemental Table 2.** Relapse rate, time to first relapse, and dose of PSL at relapse according to the initial dose

|  | Total | Group L | Group H | p value |
| --- | --- | --- | --- | --- |
| Cases in remission | (n = 87) | (n = 44) | (n = 43) |  |
| Relapse rate, n (%) | 41 (47.1) | 20 (45.4) | 21 (48.8) | 0.75 |
|  |  |  |  |  |
| Cases in relapse | (n = 41) | (n = 20) | (n = 21) |  |
| Time to first relapse from complete response, months | 15.1 [8.2–28.8] | 16.5 [7.9–29.4] | 13.7 [7.7–28.8] | 0.98 |
| Dose of PSL at relapse, mg/kg | 0.05 [0–0.15] | 0.06 [0–0.14] | 0.05 [0–0.19] | 0.82 |
| Dose of PSL at first relapse, mg | 3.75 [0–10] | 4.4 [0.3–9.4] | 3.0 [0–12.5] | 0.83 |
| Cumulative dose of PSL until first relapse, mg | 6590 [5154–10421] | 6305 [5118–8978] | 7879 [4893–11728] | 0.40 |
| Cumulative dose of PSL until first relapse, mg/kg | 102.8 [74.4–168.6] | 90.4 [65.4–121.3] | 140.8 [83.5–217.5] | 0.045 |
| Cumulative dose of PSL until first relapse, mg/kg/day | 0.23 [0.15–0.34] | 0.19 [0.13–0.26] | 0.26 [0.19–0.46] | 0.048 |

PSL, prednisolone.

Values are given as n (%) or median [interquartile range], as appropriate.

**Supplemental Table 3.** Hazard ratios with 95% confidence intervals from multivariable regression analyses of the association of the initial dose of prednisolone (Group L versus Group H) with relapse

|  | Variables included in regression models | HR (95% CI) | p value |
| --- | --- | --- | --- |
| Crude |  | 0.99 (0.53–1.84) | 0.98 |
|  |  |  |  |
| Model 1 | gender, age (per 1 year) | 0.86 (0.43–1.72) | 0.66 |
|  |  |  |  |
| Model 2 | Model 1 | 0.87 (0.44–1.75) | 0.70 |
|  | + Serum creatinine (mg/dL) |  |  |
|  | proteinuria (per 1.0 g/day) |  |  |
|  |  |  |  |
| Model 2’ | Model 1 | 0.88 (0.44–1.76) | 0.71 |
|  | + eGFR (per 1 mL/min/1.73 m^2^) |  |  |
|  | proteinuria (per 1.0 g/day) |  |  |

HR, hazard ratio; CI, confidence interval; eGFR, estimated glomerular filtration rate.

**Supplemental Table 4.** Incidence of adverse events according to the initial PSL dose

|  | Total | Group L | Group H | p value |
| --- | --- | --- | --- | --- |
|  | (n = 91) | (n = 45) | (n = 46) |  |
| Within 6 months | (n = 85) | (n = 41) | (n = 44) |  |
| Infectious complication, n (%) | 5 (5.9) | 3 (7.3) | 2 (4.6) | 0.59 |
| AKI, n (%) | 48 (56.5) | 26 (63.4) | 22 (50.0) | 0.21 |
| Death, n (%) | 3 (3.5) | 1 (2.4) | 2 (4.6) | 0.60 |
|  |  |  |  |  |
| Within 1 year | (n = 80) | (n = 40) | (n = 40) |  |
| Infectious complication, n (%) | 5 (6.3) | 3 (7.5) | 2 (5.0) | 0.64 |
| AKI, n (%) | 47 (58.8) | 26 (65.0) | 21 (52.5) | 0.26 |
| Death, n (%) | 3 (3.8) | 1 (2.5) | 2 (5.0) | 0.56 |
|  |  |  |  |  |
| Within 2 years | (n = 63) | (n = 34) | (n = 29) |  |
| Infectious complication, n (%) | 4 (6.3) | 2 (5.9) | 2 (6.9) | 0.87 |
| AKI, n (%) | 38 (60.3) | 22 (64.7) | 16 (55.2) | 0.44 |
| Death, n (%) | 3 (4.8) | 1 (2.9) | 2 (6.9) | 0.46 |
|  |  |  |  |  |
| Within entire observational period | (n = 91) | (n = 45) | (n = 46) |  |
| Infectious complication, n (%) | 10 (11.0) | 7 (15.6) | 3 (6.5) | 0.16 |
| AKI, n (%) | 52 (57.1) | 28 (62.2) | 24 (52.2) | 0.33 |
| Death, n (%) | 6 (6.6) | 4 (8.9) | 2 (4.4) | 0.38 |

AKI, acute kidney injury; PSL, prednisolone. Values are given as n (%).

**Supplemental Table 5.** Multivariable regression analyses (adjusted for gender, age (per year), serum creatinine (mg/dL), proteinuria (per 1.0 g/day), and BMI) of the association of the initial dose of prednisolone (less than 40 mg vs. 40 mg to 45 mg vs. 45 mg or more) with remission

| Comparison of groups by prednisolone dose | HR (95% CI) | p value |
| --- | --- | --- |
| less than 40 mg/45 mg or more | 0.71 (0.28–1.80) | 0.48 |
| less than 40 mg/40 mg to 45 mg | 0.96 (0.52–1.76) | 0.90 |
| 40 mg to 45 mg/45 mg or more | 0.74 (0.33–1.64) | 0.46 |

HR, hazard ratio; CI, confidence interval.

**Supplemental Table 6.** Multivariable regression analyses of the association of the initial dose of prednisolone (per 10 mg/day) with remission

|  | Variables included in the regression models | HR (95% CI) | p value |
| --- | --- | --- | --- |
| Crude |  | 1.00 (0.99–1.01) | 0.98 |
|  |  |  |  |
| Model 1 | Gender, age (per year) | 1.00 (0.99–1.00) | 0.46 |
|  |  |  |  |
| Model 2 | Model 1 | 1.00 (0.99–1.01) | 0.74 |
|  | + Serum creatinine (mg/dL) |  |  |
|  | proteinuria (per 1.0 g/day) |  |  |
|  |  |  |  |
| Model 3 | Model 2 | 1.00 (0.99–1.01) | 0.75 |
|  | + BMI |  |  |

HR, hazard ratio; CI, confidence interval.

**Supplemental Table 7.** Multivariable regression analyses (adjusted for gender, age (per year), serum creatinine (mg/dL), proteinuria (per 1.0 g/day), and BMI) of the association of the initial dose of prednisolone (less than 40 mg vs. 40 mg to 45 mg vs. 45 mg or more) with relapse

| Comparison of groups by prednisolone dose | HR (95% CI) | p value |
| --- | --- | --- |
| less than 40 mg/45 mg or more | 0.60 (0.16–2.18) | 0.43 |
| less than 40 mg/40 mg to 45 mg | 0.65 (0.25–1.70) | 0.38 |
| 40 mg to 45 mg/45 mg or more | 0.90 (0.31–2.58) | 0.90 |

HR, hazard ratio; CI, confidence interval.

**Supplemental Table 8.** Multivariable regression analyses of the association of the initial dose of prednisolone (per 10 mg/day) with relapse

|  | Variables included in the regression models | HR (95% CI) | p value |
| --- | --- | --- | --- |
| Crude |  | 1.00 (0.99–1.01) | 0.71 |
|  |  |  |  |
| Model 1 | Gender, age (per year) | 1.00 (0.99–1.01) | 0.66 |
|  |  |  |  |
| Model 2 | Model 1 | 1.00 (0.99–1.01) | 0.85 |
|  | + Serum creatinine (mg/dL) |  |  |
|  | proteinuria (per 1.0 g/day) |  |  |
|  |  |  |  |
| Model 3 | Model 2 | 1.00 (0.99–1.01) | 0.93 |
|  | + BMI |  |  |

HR, hazard ratio; CI, confidence interval.

**Supplemental Fig. 1**

Cumulative percentage of patients with relapse from first complete remission according to the initial steroid dose. There was no significant difference in the time to relapse from complete remission between Groups L and H according to the results of Kaplan–Meier analysis

Months from complete remission

0

2

4

8

16

28

Log rank

p=0.98

Cumulative % of patients with relapse

since complete remission

100

90

80

70

60

50

40

30

20

10

0

(%)

Group L

Group H
